# Virtual Care Prior to and During COVID-19: Cross-sectional Survey of Rural and Urban Adults

**DOI:** 10.2196/37059

**Published:** 2022-08-22

**Authors:** Kathy L Rush, Cherisse L Seaton, Kendra Corman, Nicole Hawe, Eric Ping Hung Li, Sarah J Dow-Fleisner, Mohammad Khalad Hasan, Nelly D Oelke, Leanne M Currie, Barbara Pesut

**Affiliations:** 1 School of Nursing University of British Columbia Kelowna, BC Canada; 2 Faculty of Management University of British Columbia Kelowna, BC Canada; 3 School of Social Work University of British Columbia Kelowna, BC Canada; 4 Department of Computer Science University of British Columbia Kelowna, BC Canada; 5 Rural Coordination Centre of British Columbia Vancouver, BC Canada; 6 Department of Community Health Sciences Cummings School of Medicine University of Calgary Calgary, AB Canada; 7 School of Nursing University of British Columbia Vancouver, BC Canada

**Keywords:** virtual care, rural, urban, COVID-19, digital literacy, unmet needs

## Abstract

**Background:**

To reduce person-to-person contact, the COVID-19 pandemic has driven a massive shift to virtual care. Defined as the use of technology (synchronous or asynchronous) to support communication between health care providers and patients, rural-urban differences in virtual care are relatively unexplored.

**Objective:**

The 2-fold purpose of this study was to examine rural and urban virtual care access, use, and satisfaction during the pandemic and to identify any unmet needs.

**Methods:**

This study was a cross-sectional online survey exploring virtual care among rural and urban adults in summer 2021 using a combination of fixed and open-ended response options. Quantitative data were analyzed using both descriptive and inferential statistics, and qualitative data were analyzed using inductive thematic content analysis.

**Results:**

Overall, 501 (373, 74.4% female; age range 19-86 years; 237, 47.3% rural-living) Western Canadians completed the survey. Virtual care use was high among both rural (171/237, 72.2%) and urban (188/264, 71.2%) participants, with over one-half (279/501, 55.7%) reporting having only started to use virtual care since the pandemic. The self-reported need for mental health programs and services increased during the pandemic, compared with prior for both rural and urban participants. Among virtual care users, interest in its continuation was high. Our analysis also shows that internet quality (all *P*<.05) and eHealth literacy (all *P*<.001) were positively associated with participants’ perceptions of virtual care usefulness, ease of use, and satisfaction, with no rural-urban differences. Rural participants were less likely to have used video in communicating with doctors or health care providers, compared with urban participants (*P*<.001). When describing unmet needs, participants described a (1) lack of access to care, (2) limited health promotion and prevention options, and (3) lack of mental health service options.

**Conclusions:**

The increased demand for and use of virtual care may reflect increased availability and a lack of alternatives due to limited in-person services during the COVID-19 pandemic, so a balance between virtual care and in-person care is important to consider postpandemic. Further, ensuring availability of high-speed internet and education to support patients will be important for providing accessible and effective virtual care, especially for rural residents.

## Introduction

The World Health Organization declared COVID-19 a pandemic and global health emergency on March 11, 2020 [1]. Respecting no geographic boundaries, the pandemic has impacted both rural and urban populations. However, the pandemic was superimposed on well known urban-rural health and health service disparities. Compared with their urban counterparts, rural dwellers experience poorer health and health behaviors, more chronic conditions, and shorter life expectancies and higher mortality rates [2]. These health inequities are systemic and avoidable differences in health that are caused by the unfair distribution of resources, wealth, and power in society [3] and reflect social and structural determinants of health, such as educational, financial, social, and geographical difficulties [4,5]. Moreover, rural communities have historically lacked access to health services and care, due to heightened health provider shortages, underdeveloped digital infrastructure, travel burdens, and costs [6,7].

Adding to the existing social-structural factors influencing rural health and health service use, the pandemic has contributed further to health care gaps [3]. The deferral of elective procedures and routine checkups [8,9] and patient avoidance of medical care for non-COVID-19 illness due to fear of contracting the virus [10] accounted for a massive global reduction in health care utilization (by one-third) during COVID-19 [8]. At the same time, a meta-analysis of 60 studies reported high levels of depression and anxiety worldwide [11], generating high demand for mental health services that have been disrupted by COVID-19 [12]. Few studies have considered the extent of unmet health and wellness needs as a result of these gaps and whether there are urban and rural differences. In their South Korean study [13], researchers found that demographics (eg, age, sex, educational level), chronic diseases, and stress and anxiety were associated with unmet care. To prevent or reduce exposure with the emergence of new variants coupled with the redirection of resources for COVID-19 patients, health care was drastically altered. COVID-19 catalyzed a massive shift to virtual care, with reported increases as much as 56-fold compared with prior to COVID-19 [14,15]. The term virtual care is often used interchangeably with telemedicine or telehealth; we are referring specifically to virtual care defined as the use of technology (synchronous or asynchronous) to support communication between health care providers and patients [16]. Virtual care technologies include video visits, email, text messaging, and telephone visits. In an online survey in summer 2020, 31% of rural adults reported using virtual care somewhat and far more often after March 2020 [17]. However, in a Canadian rural-urban comparison study using an administrative database, urban uptake of virtual care increased at a steeper rate than rural uptake at the start of the pandemic (220 vs 147 visits per 1000 patients) [18]. Similarly, 53% of US urban households, compared with 46% of rural households, used virtual care during the pandemic, though this difference was not statistically significant [19]. In their US study of rural and urban living veterans, Hogan and colleagues [20] found higher use of virtual mental health care pre-COVID-19 among rural than among urban living veterans and increased use by both groups during the first 7 months into COVID-19 but with urban veterans surpassing rural veterans’ usage. Researchers have suggested that barriers to a rapid transition to telehealth delivery may have affected rural areas more than urban areas particularly due to their lack of access to broadband internet, limited device ownership (smartphone, tablet, laptop), and lower digital literacy [20].

Despite lower uptake of virtual care, satisfaction with virtual care was high among rural-dwelling individuals both before [21] and during COVID-19 [22]. In a US study, virtual care satisfaction was higher in rural (88%) than in urban (84%) areas, though not significantly different [19]. Whether satisfaction translates into willingness to continue virtual care postpandemic deserves more research; however, in a recent COVID-19 study of 1059 US residents, 72% to 77% reported intentions to continue to use virtual care, at least for acute health conditions, with no rural-urban differences [23].

Another factor that impacts users’ ability to use, and satisfaction with, virtual care is eHealth literacy, which is defined as the ability to find, use, and apply health information from electronic sources [24]. Although inextricably linked to rural challenges in access to high-speed internet, eHealth literacy is often reported to be lower among rural residents compared with their urban counterparts [25]. In a small US study (n=253), utilization of and satisfaction with virtual care were associated with higher eHealth literacy among rural-living adults [22]. Similarly, a significantly positive relationship was found between eHealth literacy and satisfaction with virtual care among people living peripherally to Israel [26].

Given the massive impact of COVID-19 on virtual care, a comprehensive examination of rural and urban virtual care access, use, satisfaction, and future intention to use, considering eHealth literacy and unmet needs, is needed. No such study has been conducted at the time of this study—a full year after COVID-19 was declared a pandemic [1]. The purpose of this study was to compare rural- and urban-living Canadian adults’ access, use, satisfaction, and intentions to continue to use virtual care, as well as to explore unmet health and wellness needs 1 year after COVID-19 was declared a pandemic.

## Methods

### Study Design, Context, and Participant Recruitment

This study employed a cross-sectional online survey open to adults (19 years or older) residing in urban, rural, and remote communities in a Western Canadian province where 18.44% live in rural communities [27]. The online survey was open to participants for a 6-week period (June 24, 2021, to August 9, 2021). During this time, the province was in a state of re-opening [28]. Step one of the provincial re-start plan began May 25, 2021; social restrictions were loosened, businesses re-opened, and recreational activities resumed. Step two of the re-start (from June 15, 2021, to June 30, 2021) included additional lifting of travel restrictions and easing of restrictions for businesses and recreational activities (eg, liquor served until midnight, up to 50 spectators at outdoor sporting events allowed). The COVID-19 vaccine was available to everyone age 12 years and older during the time of this survey [29].

Recruitment efforts primarily involved Facebook posts targeting local community pages (n=35; eg, “What’s Up [community name]”) together totaling over 177,000 members as well as through 3 paid Facebook advertisements (“post boosts”) targeting adults living within a 25-mile radius of several rural and urban communities in the province. Email invitations with the survey link were also sent to rural-living participants who completed an online survey in 2020 [17] and consented to being contacted for a future survey. Advertisements were also posted on Twitter, Kijiji (Canadian Craigslist), Facebook, and rural websites and in the volunteer sections of classified web pages, as well as shared through targeted announcements in rural community association newsletters. Additionally, REACH BC, an online platform designed to connect individuals across British Columbia (BC) with research opportunities; Patient Voices Network, a partner platform of REACH BC; and a network aimed at engaging patients in their health care were used for advertisement and recruitment. Although we were unable to track how many potential respondents were reached in total, the 3 Facebook advertisements had a combined estimated audience reach of 5776 adults and engagement (link clicks) of 109 (1.9% response rate), and 56 of the 206 (27.2%) previous survey participants completed this survey. Due to more individuals residing in urban areas than in rural communities, it was anticipated that recruitment of urban participants would be more efficient. Accordingly, more recruitment efforts were focused on targeting participants in rural communities. To promote participation, 5 CAD $100 (US $77.61), 3 CAD $200 (US $155.22), and 1 CA $400 (US $310.43) draw prize incentives were advertised. The survey used a combination of fixed and open responses and an attention check question (“If you are a human reading this, please select strongly agree”) to detect survey bots and inattentive respondents [30,31].

### Ethical Considerations

The study was conducted in accordance with the Declaration of Helsinki and with the Canadian Tri-Council Policy Statement. All participants provided informed consent online prior to completing the survey. Participants were provided a link to download the consent form and encouraged to keep a copy for their personal records. Consent was obtained by participants selecting “Yes” in response to the question “Do you consent to participate?” This study was reviewed and received ethics approval from the University of British Columbia—Okanagan Behavioural Research Ethics Board (H20-01166).

### Measures

#### Rurality

Participants provided their community’s name, and based on the census subdivision of the community, a score was assigned from Statistics Canada’s Index of Remoteness [32]. Remoteness index scores are based on population size and cost to travel to the nearest population center and range from 0 to 1, with scores closer to 1 indicating greater remoteness. Based on the manual method of classification into 5 categories of accessibility using predetermined cutoffs by Subedi et al [33], community scores categorized as easily accessible (<0.1500) or accessible (0.1500 to 0.2888) were classified as urban, and community scores categorized as less accessible (0.2889 to 0.3898), remote (0.3899 to 0.5532), or very remote (>0.5532) were classified as rural.

#### Demographic Characteristics of Participants

Demographic data collected from all participants included age in years (open response), gender (female, male, nonbinary, prefer not to answer, other), ethnicity/race (select all from options provided or enter under “other”), education (response options ranging from “some high school or less” to “university degree”), and occupation (working full-time, working part-time, going to school, retired, not employed, other).

#### General Health and Health Care Service Use

Participants were asked to rate their health on a scale ranging from poor (1) to excellent (5). They were also asked to indicate the frequency of contact with their doctor or health care provider in the last 12 months (never, once, 2-5 times, 6-11 times, or 12 or more times) and whether their communications with their doctor or health care provider during COVID-19 had included a series of video or nonvideo interfaces (select all that apply). Those who selected video alone or along with other modes of communication were grouped as having used video, and those who did not select video were grouped as having not used video.

#### Virtual Care Use and Satisfaction

Participants were provided with a definition of virtual care as using technology (including email, text messaging, video visits, and telephone visits) to communicate with clinicians. Participants were then asked about their engagement with virtual care, with the question “Have you used virtual care?” Response options included: “Yes, and I used virtual care prior to the COVID-19 pandemic,” “Yes, but I only began using virtual care since the COVID-19 pandemic began,” and “No.” Those who responded “Yes” were asked to complete modified 5-point (strongly disagree to strongly agree) subscales of the Telehealth Usability Questionnaire (TUQ) [34] related to Usefulness, Ease of use, and Satisfaction. The TUQ was modified to specifically refer to “virtual care” instead of “telehealth.” Mean subscale scores were calculated, with a higher score indicating greater usefulness, ease of use, and satisfaction with virtual care. The TUQ has strong content validity and good to excellent internal consistency [34]. In this study, Cronbach alphas were .82 for Usefulness, .84 for Ease of use, and .90 for Satisfaction subscales.

#### Intention to Use Virtual Care Postpandemic

The participants who reported using virtual care were also asked to answer a 4-item, 7-point (strongly disagree to strongly agree) scale about their intention to use virtual care postpandemic [23], modified to remove reference to “acute” conditions. Confirmatory factor analysis, internal reliability, and construct reliability have been reported [23]. In the present study, the Cronbach alpha was .86 for the future intentions to use virtual care scale.

#### Health Service Need and Access

A series of questions asking participants about their health service needs and access both before and during COVID-19 were generated based on the expertise of the research team and a previous survey suggesting that mental health needs might be a focal area, given the impact of COVID-19 [35]. Health services included virtual care, online mental health programs, and video or phone mental health services (eg, connecting with someone). Participants were asked to indicate if they “needed and had access to,” “needed and did not have access to/not aware if available,” or “did not need” these services both before and during COVID-19. Responses were then grouped into “needed” versus “not needed” for comparison. In addition, an open-ended question, “Please describe any unmet health or wellness needs you have had since the start of the COVID-19 pandemic (March 2020),” explored unmet needs.

#### eHealth Literacy

Participants completed an 8-item, 5-point (strongly disagree to strongly agree; eg, “I know what health resources are available on the internet”) electronic Health Literacy Scale (eHEALS) [24] that measures “combined knowledge, comfort, and perceived skills at finding, evaluating, and applying electronic health information to health problems.” Summative scores range from 8 to 40. Previous research using the eHEALS has demonstrated moderate test-retest reliability, good internal consistency, and construct validity [24,35]. In this study, the Cronbach alpha was .92 for the eHEALS scale.

#### Internet Quality

Participants were asked to rate the adequacy of their internet access during their day-to-day life on a scale ranging from 1 to 7, where 1 represents poor/inadequate (minimal to no reliability and very poor quality) and 7 represents excellent/adequate (always reliable and high quality).

### Analysis

Descriptive statistics (frequencies; means and SDs) were used to summarize the data. Chi-square tests were used to examine participant characteristics and virtual care use by rurality (rural or urban). Independent samples *t* tests were used to investigate differences in categorical variables (ie, rural vs urban and used video vs telephone only) on virtual care usefulness, ease of use, satisfaction, future use, eHealth literacy, and internet quality. Regression analyses were used to examine relationships between virtual care scale scores and age, general health, eHealth literacy, internet adequacy, and remoteness. Normality was examined using histograms and P-P plots. Internet adequacy and age were slightly skewed but considered acceptable given the large sample size. Multivariate outliers and influential cases were examined using casewise diagnostics, Cooks distance, Mahalanobis distance, and leverage. Two cases consistently came up as outliers in each regression analysis; however, these were not consistently unusual on other variables, and regression results were the same with and without these cases, so they were retained. All regression analyses met assumptions of linearity, heteroscedasticity, and multicollinearity. Quantitative data were analyzed using SPSS version 27 (IBM Corp, Armonk, NY) [36]. Open-ended responses were analyzed by 2 research team members (CLS, KC), and inductive thematic analysis was used to code and determine central themes. Two research team members independently coded the responses. Once initial coding was completed, similar codes were clustered into derived themes using consensus. NVivo 12 (QSR International, Burlington, MA) was used to analyze the qualitative data.

### Data Screening

Over the 6-week data collection period, 617 total responses were collected, 116 of which were excluded due to the participant selecting “under 19 years” or “not a resident of [Province]” in response to initial eligibility questions and exit survey (n=39), survey incompletion beyond demographics (n=33), unidentified community name of residence (n=6), inattentive and inaccurate responses (n=8), or survey bots (n=30). An attention check question is a moderately effective strategy for survey bot detection, but other factors were considered (eg, repeating same response options across multiple questions, similar illogical responses to open-ended questions, unrealistic survey completion times) in determining the exclusion of potential survey bots [31]. After removing the exclusions, the final sample of 501 (373/501, 75.0% female) responses were retained for the current study and analyses.

## Results

### Participant Characteristics

In total, 237 (237/501, 47.3%) participants were classified as rural-living, and 264 (264/501, 52.7%) were urban-living. Characteristics of the sample are presented in Table 1. Urban participants (mean age 34.8 years) were, on average, younger than rural participants (mean age 48.8 years). Rural participants were more likely to have trades certification/diploma, while urban participants were more likely to be working or going to school and less likely to be retired or unemployed. Rural participants were more likely to be Indigenous or Caucasian (218/237, 92.0%), whereas urban participants were more likely to be Caucasian or Asian or mixed ethnicity (217/264, 82.2%). There were no rural/urban differences in general health or number of health care visits over the past year, though rural participants were less likely to have used video in communicating with health care providers, compared with urban participants.

**Table 1:** Characteristics of all (n=501), rural (n=237), and urban (n=264) participants.

Characteristics			All participants, n (%)		Rural, n (%)		Urban, n (%)		*χ*^2^ (df)		*P* value^a^
**Age (range: 19-86 years)**											
	19-35 years	238 (47.5)		64 (27.0)		174 (65.9)		78.5 (2)		<.001	
	36-54 years	117 (23.4)		69 (29.1)		48 (18.2)					
	≥55 years	142 (28.3)		101 (42.6)		41 (15.5)					
	Missing/prefer not to answer	4 (0.8)		3 (1.3)		1 (0.4)					
**Gender**											
	Female	373 (74.5)		179 (75.5)		194 (73.5)		0.4 (1)		.52^b^	
	Male	121 (24.2)		54 (22.8)		67 (25.4)					
	Nonbinary	6 (1.2)		4 (1.7)		2 (0.8)					
	Prefer not to answer	1 (0.2)		0 (0)		1 (0.4)					
**Education**											
	Some high school or less	16 (3.2)		12 (5.1)		4 (1.5)		21.6 (3)		<.001	
	Completed high school	126 (25.1)		49 (20.7)		77 (29.2)					
	Trades certification/diploma	124 (24.8)		77 (32.5)		47 (17.8)					
	University degree	231 (46.1)		98 (41.4)		133 (50.4)					
	Missing/prefer not to answer	4 (0.8)		1 (0.4)		3 (1.1)					
**Ethnicity**											
	Indigenous (First Nation/Inuit/Metis)	26 (5.2)		19 (8.0)		7 (2.7)		92.1 (4)		<.001	
	Asian (including South/Southeast)	94 (18.8)		7 (3.0)		87 (33.0)					
	Caucasian/White	321 (64.1)		191 (80.6)		130 (49.2)					
	Indigenous and Caucasian	16 (3.2)		8 (3.4)		8 (3.0)					
	Other/mixed ancestry	37 (3.2)		10 (4.2)		27 (10.2)					
	Missing/prefer not to answer	7 (1.4)		2 (0.8)		5 (1.9)					
**Occupation**											
	Working or going to school	356 (71.1)		155 (65.4)		201 (76.1)		8.8 (1)		.003	
	Retired or not employed	137 (27.3)		80 (33.8)		57 (21.6)					
	Missing/prefer not to answer	8 (1.6)		2 (0.8)		6 (2.3)					
**General health**											
	Poor	21 (4.2)		8 (3.4)		13 (4.9)		1.2 (4)		.88	
	Fair	70 (14.0)		33 (13.9)		37 (14.0)					
	Good	173 (34.5)		81 (34.2)		92 (34.8)					
	Very good	183 (36.5)		86 (36.3)		97 (36.7)					
	Excellent	46 (9.2)		24 (10.1)		22 (8.3)					
	Missing/prefer not to answer	8 (1.6)		5 (2.1)		3 (1.1)					
**Health care provider visits (past 12 months)**											
	Never	34 (6.8)		15 (6.3)		19 (7.2)		2.6 (4)		.63	
	Once	61 (12.2)		27 (11.4)		34 (12.9)					
	2-5 times	247 (49.3)		115 (48.5)		132 (50.0)					
	6-11 times	98 (19.6)		53 (22.4)		45 (17.0)					
	≥12 times	51 (10.2)		22 (9.3)		29 (11.0)					
	Missing/prefer not to answer	10 (2.0)		5 (2.1)		5 (1.9)					
**Communication with health care providers**											
	Used video	162 (32.3)		58 (24.5)		104 (39.4)		12.7 (1)		<.001	
	Did not use video	339 (67.7)		179 (75.5)		160 (60.6)					

^a^Chi-square tests comparing rural with urban participants.

^b^Chi-square for gender only compared men with women due to expected counts falling below 5 for the other categories.

### Virtual Care

Virtual care use was high, with over one-half (279/501, 55.7%) of participants reporting having only started to use virtual care since the onset of the COVID-19 pandemic (see Table 2). The pattern of virtual care use was not different for rural versus urban participants (^2^_2_=1.03, *P*=.60). There were more female users than male users of virtual care (^2^_2_=14.92, *P*=.002). There were no age differences in virtual care use or nonuse.

The need for virtual health care and for mental health programs and services increased significantly during the COVID-19 pandemic compared with prior to COVID-19 (see Table 3). This pattern of increased need was similar in both rural and urban participants. A greater proportion of urban participants reported needing all mental health services both pre- and during COVID-19, though the only significant difference was that urban participants had a greater need for online mental health programming pre-COVID-19 (^2^_1_=5.08, *P*=.02).

**Table 2:** Virtual care use among all (n=501), rural (n=237), and urban (n=264) participants.

Virtual care use	Total, n (%)	Rural, n (%)	Urban, n (%)
Has not used virtual care	142 (28.3)	66 (27.8)	76 (28.8)
Has used virtual care and used virtual care prior to COVID-19	80 (16.0)	42 (17.7)	38 (14.4)
Has used virtual care but only since the onset of COVID-19 (March 2020)	279 (55.7)	129 (54.4)	150 (56.8)

**Table 3:** Comparison of services needed versus not needed between rural (n=237) and urban (n=264) participants, pre-COVID-19 and post-COVID-19.

Services needed			Before COVID-19				During COVID-19				*χ*^2^ (df)		*P* value^a^
			Rural, n (%)		Urban, n (%)		Rural, n (%)		Urban, n (%)				
**Virtual care**													
	I needed	72 (30.4)		78 (29.5)		146 (61.6)		171 (64.8)		58.4 (1)		<.001	
	I did not need	134 (56.5)		160 (60.6)		54 (22.8)		56 (21.2)					
	Missing	31 (13.1)		26 (9.8)		37 (15.6)		37 (14.0)					
**Online mental health programs**													
	I needed	40 (16.9)		69 (26.1)		61 (25.8)		89 (33.8)		209.9 (1)		<.001	
	I did not need	163 (68.8)		169 (64.0)		134 (56.5)		138 (52.3)					
	Missing	34 (14.3)		26 (9.8)		42 (17.7)		37 (14.0)					
**Video or phone mental health services**													
	I needed	51 (21.3)		76 (28.9)		80 (33.7)		107 (40.5)		163.1 (1)		<.001	
	I did not need	154 (65.0)		162 (61.4)		115 (48.5)		118 (44.7)					
	Missing	32 (13.5)		26 (9.8)		42 (17.7)		39 (14.8)					

^a^Chi-square tests comparing needed versus not needed before versus during COVID-19 (ie, collapsed across rural and urban participants).

Among those who “needed” virtual care, online mental health programs and phone or video mental health services, we also explored the proportion who either did not have access to the services or were not sure if the services were available (see Table 4). There were no differences in the pattern of results by rural versus urban, so combined data are presented for simplicity. Before COVID-19, for both rural and urban participants, there was a higher percentage who needed the services or programs and did not have access compared with those who needed them but did have access to all services or programs. Then, during COVID-19, the numbers evened out or even reversed—where a significantly higher percentage needed and had access, compared with needed and did not have access, across both rural and urban participants.

**Figure 1:**
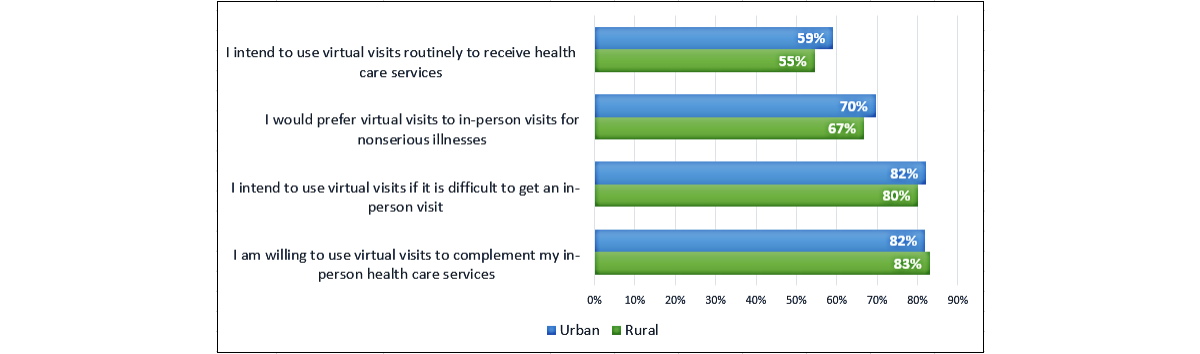
Proportion of rural versus urban participants who agreed, somewhat agreed, or strongly agreed with the intention to continue to use virtual care items. This is the proportion among only those who had used virtual care previously. About 28% (142/501) of the sample had not used virtual care and therefore did not answer these questions.

There were no rural-urban differences in virtual care usefulness, ease of use, satisfaction, intention to use in future, or eHealth literacy; however, internet quality was reported to be significantly worse among rural participants than among urban participants (see Table 5). There were no gender differences on virtual care scale scores, eHealth literacy, or internet adequacy. Those who had used video had significantly higher scores for virtual care usefulness, ease of use, satisfaction, intention to use in future, eHealth literacy, and internet adequacy scores compared with those who had not used video (see Table 6). There was a great deal of interest in continuing to use virtual care postpandemic (see Figure 1), with no significant rural-urban differences.

When controlling for age and general health, eHealth literacy and internet adequacy were positively associated with all virtual care usefulness, ease of use, and satisfaction scores (see Table 7). Only eHealth literacy individually contributed to future intentions to use virtual care when all predictors were entered in the model simultaneously.

**Table 4:** Comparison of access versus no access pre- to post-COVID-19 among those who needed virtual care, online mental health programs, and phone or video mental health services.

Services needed			Before COVID-19, n (%)		During COVID-19, n (%)		*χ*^2^ (df)		*P* value^a^
**Virtual care**									
	Needed and had access to	63 (12.6)		290 (57.9)		5.5 (1)		.03	
	Needed but did not have access or was unaware	87 (17.4)		27 (5.4)					
**Online mental health programs**									
	Needed and had access to	31 (6.2)		71 (14.2)		25.0 (1)		<.001	
	Needed but did not have access or was unaware	78 (15.6)		79 (15.8)					
**Video or phone mental health services**									
	Needed and had access to	55 (11.0)		136 (27.1)		28.9 (1)		<.001	
	Needed but did not have access or was unaware	72 (14.4)		51 (10.2)					

^a^Chi-square tests comparing pre- with during COVID-19.

**Table 5:** Rural and urban participant scores on virtual care scales, eHealth literacy, and internet adequacy.

Scores		Rural (n=171), mean (SD)	Urban (n=188), mean (SD)	*t* test (df)	2-sided *P* value^a^
**Virtual care**					
	Usefulness (range 1-5)	3.8 (0.87)	3.89 (0.82)	1.00 (357)	.32
	Ease of use (range 1-5)	3.79 (0.74)	3.90 (0.77)	1.48 (357)	.14
	Satisfaction (range 1-5)	3.83 (0.86)	3.88 (0.82)	0.47 (356)	.64
	Future intentions to use (range 1-7)	5.03 (1.38)	5.23 (1.37)	1.36 (350)	.17
eHealth literacy (range 1-5)		3.91 (0.72)	3.91 (0.96)	0.04 (485)	.97
Internet adequacy (range 1-7)		5.47 (1.57)	5.98 (0.07)	4.01 (485)	<.001

^a^*t* tests comparing rural with urban.

**Table 6:** Virtual care scale, eHealth literacy, and internet adequacy scores of those who had used video versus those who had not.

Scores			Used video (n=203), mean (SD)		Had not used video (n=156), mean (SD)		*t* test (df)		2-sided *P* value^a^
**Virtual care**									
	Usefulness (range 1-5)	4.08 (0.73)		3.67 (0.89)		4.64 (357)		<.001	
	Ease of use (range 1-5)	4.04 (0.75)		3.70 (0.73)		4.39 (357)		<.001	
	Satisfaction (range 1-5)	4.10 (0.76)		3.67 (0.85)		5.03 (356)		<.001	
	Future intentions to use (range 1-7)	5.53 (1.35)		4.82 (1.33)		4.90 (350)		<.001	
eHealth literacy (range 1-5)			4.08 (0.65)		3.83 (0.71)		3.82 (485)		<.001
Internet adequacy (range 1-7)			5.96 (1.34)		5.64 (1.41)		2.43 (485)		.02

^a^*t* tests comparing video users with non-video users.

**Table 7:** Regression analyses examining the association between predictors age, general health, eHealth literacy, internet adequacy, and remoteness with virtual care scale scores (outcomes).

Scores			Β^a^		Coefficient *P* value		Overall R^2^		*F* test (df)		Model *P* value^b^
**VC^c^ usefulness**											
	Age	–.13		.03		0.11		8.55 (5,336)		<.001	
	General health	.03		.59							
	eHealth literacy	.24		<.001							
	Internet adequacy	.12		.04							
	Remoteness (RI^d^)	.05		.37							
**VC ease of use**											
	Age	–.10		.07		0.15		11.80 (5,336)		<.001	
	General health	.05		.32							
	eHealth literacy	.29		<.001							
	Internet adequacy	.12		.03							
	Remoteness (RI)	.02		.71							
**VC satisfaction**											
	Age	–.02		.77		0.09		6.70 (5,335)		<.001	
	General health	.01		.86							
	eHealth literacy	.24		<.001							
	Internet adequacy	.12		.047							
	Remoteness (RI)	.03		.66							
**Future intentions to use VC**											
	Age	–.06		.29		0.10		7.53 (5,330)		<.001	
	General health	–.03		.57							
	eHealth literacy	.30		<.001							
	Internet adequacy	.06		.27							
	Remoteness (RI)	.01		.89							

^a^Standardized beta coefficients (β) are reported.

^b^All 5 predictors were entered simultaneously in separate regression analyses for each virtual care outcome.

^c^VC: virtual care.

^d^RI: remoteness index.

### Unmet Needs

#### Themes

Of the 501 participants, 294 (58.7%) responded to the unmet needs open-ended question. The proportion of the rural participants who responded to the question and reported “no unmet needs” (53/132, 40.2%) was similar to the proportion of the urban participants with “no unmet needs” (58/162, 35.8%). For the comments reporting unmet needs, thematic analysis was used to construct 3 inter-related main themes: (1) lack of access to desired care, (2) limited health promotion and prevention options, and (3) mental health impacts and service adequacy or options.

#### Lack of Access to Desired Care

Among those describing unmet health or wellness needs, both rural and urban participants described reduced or limited access to desired care due to restricted or delayed in-person care (appointments, services), lack of virtual communication options to accommodate disability, and care avoidance from personal fears of viral exposure. Underlying their desire for different care were participants’ concerns or anxieties about aspects of their care being missed. A 30-year-old rural participant described her response to delayed in-person care:

Being pregnant, I was unable to see a health care provider in person before 20 weeks. This led to a lot of anxiety and I felt I didn’t have proper prenatal care.

In some cases, participants reported serious consequences, such as a 51-year-old urban participant’s “ruptured appendix was undiagnosed and ended up in emergency; lucky to be alive.” Other participants were unwilling or afraid to access care (delaying care), as an urban participant described: “Visiting doctor or hospital involves risk of exposure to virus.”

Participants also expressed that new virtual care platforms did not address the needs of individuals with disabilities. One 48-year-old urban participant shared her challenges in using virtual care due to the temporary loss of her voice:

I lost my voice during the pandemic and have difficulty speaking, due to what we now know are post-acute Covid-19 syndrome neurologic issues. I needed access to texting, emailing, chatting communication options with medical providers because I could not speak, or be understood when calling. I literally could not call 911 for help because at times I could not communicate using speech. My primary care physician refuses to use virtual meetings, whereby I would have been able to at least use the chat function to communicate. This situation has caused me panic attacks, isolation from medical care, and other mental health issues rooted in hopelessness and fear.

#### Limited Health Promotion and Prevention Options

Participants also reported that less critical health promotion and prevention options had been greatly reduced, such as delays in routine health checking, cancer screenings, and dental appointments. A 58-year-old rural participant explained:

I have not been able to schedule my routine cancer screening exam (due every 3 years) with my physician. I also have not had a massage in over 18 months, and it was over a year before I went in to get my teeth cleaned.

The mandatory shutdown of health centers and gyms also impacted some urban participants’ exercise routines:

I’ve been less able to attend the gym facility [that] I used to use multiple times per week, especially with the most recent wave, and likely lost strength in my muscle groups. I’ve recently injured my knee and suspect this is the reason. As well, I’ve gained weight, likely in part for the same reason.

#### Mental Health Impacts and Service Adequacy

Finally, unmet mental health needs were indicated in response to this open-ended question. Participants described the negative socioemotional impacts of the pandemic, such as depression, anxiety, stress, and social isolation, and the corresponding lack of adequate mental health services to meet their needs. For example, one 59-year-old rural participant stated, “I have suffered from depression since the start of the pandemic,” whereas an urban participant expressed having “More anxiety and stress due to concerns about the pandemic. Especially safety of my loved ones.” Similarly, many participants expressed unmet social needs, restrictions on social gatherings, and the ever-changing COVID-19 safety protocols at work that had detrimental impacts on their mental health. One urban participant stated that “[their] mental health, specifically depression and anxiety, has deteriorated [gotten worse] since [their] ability to engage with others and make connections was limited to virtual options.”

Some participants also expressed unmet needs related to barriers to accessing mental health services, including affordable and ongoing versus crisis-oriented mental health services, and privacy concerns. One 24-year-old urban participant described her challenge with virtual privacy:

It is difficult to do online [metal health] sessions even from home when there are other people in the house (which is very often due to the pandemic).

Others felt like there were limited mental health services available, especially affordable options, as one 22-year-old urban participant explained:

Although there were mental health [services] available, I find that the majority of the free ones are crisis support, but not ongoing mental health support. I wish there were more low-cost or free ongoing counselling supports that were available, as I wasn’t necessarily always in crisis but I still needed help with my mental health.

One 27-year-old urban participant expressed that there is a need for more government-provided mental health programs:

My overall mental health took a decline with the increased expectations of my workplace and level of stress living in a small apartment with my partner. I wish that I could have more access to government-provided mental health counselling with a human, either in-person or visually

virtually

## Discussion

### Principal Findings and Comparison With Prior Work

The purpose of this study was to examine rural and urban virtual care access, use, and satisfaction during the COVID-19 pandemic, as well as to explore future intentions to use virtual care and understand participants’ unmet health and wellness needs. Virtual care use, satisfaction, and future intentions to use were all high, with no rural-urban differences. Several unmet needs were identified.

Our finding that virtual care satisfaction was high among both rural and urban participants mirrors other research [19,22] and is consistent with participants’ high levels of interest in continuing virtual care. However, it contrasts with participants in a peripheral/outlying area of Israel who had low levels of interest in continuing virtual sessions postpandemic in December 2020 [26], reflecting their low levels of satisfaction with virtual sessions (only 36%). Noteworthy were significantly higher future intentions to use virtual care among those study participants who had used video compared with those who had not. Prior to the pandemic, Ghaddar et al [37] found 78.9% of participants from an underserved Hispanic (Texas-Mexico) border community were somewhat or very likely to use telehealth services if offered. It may be that patients in underserved areas will continue to be willing to use virtual care, but the extent to which urban-dwelling patients and providers will be willing to continue virtual care may depend on the ongoing risk of exposure to the virus [38]. Yet, contrary to this notion, in a survey of South Korean urban virtual care users, fear of COVID-19 exposure was not associated with virtual care acceptance [39]. More research is needed on the role COVID-19 anxiety plays in willingness to use virtual care among both rural and urban adults.

Similar to our findings, higher use of and satisfaction with virtual care were associated with higher eHealth literacy among rural Virginians [22] and residents in peripheral areas of Israel [26]. Unlike other research that has found lower health literacy in rural populations [25], there were no rural and urban differences in eHealth literacy scores in our study population. However, the online survey was not available to those without internet or device access, and it is not clear if lack of access to internet or devices is associated with lower eHealth literacy levels. Indeed, our findings support the notion that challenges with internet quality, even among a connected sample, play a role in virtual care satisfaction in addition to eHealth literacy. Regular access to the internet was associated with higher satisfaction among rural US adults [22]. Virtual care used to its full capacity (eg, video) requires adequate broadband access [40], and more urban than rural participants in the present study reported using video virtual care. In a telephone survey of a nationally representative US sample, only 36% of rural US households without high-speed internet had used telehealth compared with 53% of households with it [19]. By including measures of not only eHealth literacy but also internet quality and rurality, we were able to discern the unique contribution of each of these characteristics to virtual care satisfaction.

The need for virtual mental health programming and services increased among both rural and urban participants during the pandemic compared with before the pandemic, but encouragingly, a higher percentage of those who needed virtual mental health programs and services had access to these during the pandemic compared with before the pandemic. However, the need for online mental health programs and services in this study was higher among urban participants than among rural participants, with urban participants’ needs significantly higher pre-COVID-19. This is consistent with pre-COVID-19 evidence indicating that risk for mental health problems was higher in urban than rural communities [41]. Urbanization and increased population density during a viral pandemic may exacerbate mental health needs and provoke greater anxiety; indeed, in a study from China, urban participants reported more severe anxiety and depression during the COVID-19 pandemic compared with rural participants [42]. It is also possible that urban participants were previously more reliant on in-person services that were less available to rural participants. Even so, mental health services and programs for special needs (eg, autism, youths, seniors, physical disabilities) are not accessible to many rural residents; therefore, it is crucial to ensure continuous mental health support to these populations. How rural and urban-dwelling adults access mental health services is a topic that requires continuing study.

Open-ended survey responses revealed that one-third or more of rural (79/237, 33.3%) and urban (104/264, 39.4%) participants in our study had a variety of unmet health and health service needs. Among these were lack of access to desired care (eg, obstetrics and gynecology visits, specialty care), delayed preventive care (eg, health checks, cancer screenings), limited health promotion and prevention options (eg, access to gyms), and lack of affordable and ongoing versus crisis-oriented mental health services. Similarly, Czeisler et al [43] found that, during the pandemic, 12% and 32% of US residents delayed or avoided urgent or ED care and routine care, respectively, because of concerns about COVID-19. Whether system- or patient-initiated, delayed care can have detrimental health consequences [44]. Additionally, a survey of 400,000 BC residents showed decreases in health-promoting behaviors (eg, exercise, healthy eating) and increases in alcohol and cannabis consumption during the pandemic [45], with likely longer-term negative population health impacts. Delayed access to care, including routine health checks and cancer screenings, has been documented. Furthermore, despite increased need for counselling, many BC residents reported an unwillingness to use virtual mental health services, viewing these as only for crisis situations [45], a notion that also surfaced in our findings. Although these themes relate to unmet needs specifically during COVID-19, a unique time of reduced service options, they provide valuable learnings for future virtual health delivery when service reductions are no longer a public health requirement.

### Limitations and Future Research

The online nature of the survey and recruitment efforts excluded the perspectives of those without access to the internet. Despite this, we had participants from very remote locations, suggesting approximation of a representative sample with respect to rurality. Further, our results were consistent with comparative evidence from both online and telephone surveys [19]. Still, the primarily social media recruitment limits the generalizability of the results. The survey was conducted during the summer months when the province was in a state of re-opening (eg, recreational activities were resuming) when a vaccine was widely available and prior to announcements of a fourth wave (Delta variant) and vaccine passports and mandates, which may have influenced responses; yet, the timing of the survey during a phase of lower risk might mean that the finding that over two-thirds of participants were willing to continue virtual care use is a conservative estimate. It should also be noted, that during the time of the survey, significant climate hazard events in the province may have impacted participation and participant mental health; for example, during June 25, 2021, through July 1, 2021, a record-breaking heat wave swept the province [46], and the subsequent wildfire season was one of the worst on record [47].

More research is needed to explore why the pattern of access among those who needed virtual mental health services might have changed; it is possible that increased programming and advertising has contributed to greater availability and awareness and perhaps less stigma. Furthermore, although this study explored virtual care access, satisfaction, and intentions, more research is needed to explore unmet in-person health care needs. The present results reinforce the notion that some things (eg, surgeries) cannot be done virtually. The trade-off between reducing exposure and delaying care will be important to consider for future research and care.

### Conclusions

Overall, findings from this study suggest that eHealth literacy and internet quality play important roles in virtual care satisfaction and future intentions to use virtual care. Yet, despite high levels of satisfaction with virtual care among both rural and urban participants, open-ended responses highlighted many unmet needs, reinforcing the notion that virtual care can supplement, but not replace, in-person care. Understanding not only use of but also rural and urban patient satisfaction with virtual care and whether patient demand will continue post-COVID-19 are important considerations for providers and policy makers. If virtual care is to be incorporated into ongoing practice following COVID-19, it is important that equitable access is addressed.

## Abbreviations

BC: British Columbia
eHEALS: electronic Health Literacy Scale
TUQ: Telehealth Usability Questionnaire
UBC: University of British Columbia
